# Composite Long- and Short-Read Sequencing Delivers a Complete Genome Sequence of B04Sm5, a Reutericyclin- and Mutanocyclin-Producing Strain of Streptococcus mutans

**DOI:** 10.1128/MRA.01067-20

**Published:** 2020-11-19

**Authors:** Jonathon L. Baker, Anna Edlund

**Affiliations:** a Genomic Medicine Group, J. Craig Venter Institute, La Jolla, California, USA; b Department of Pediatrics, University of California at San Diego, La Jolla, California, USA; University of Rochester School of Medicine and Dentistry

## Abstract

Streptococcus mutans strain B04Sm5 was recently shown to inhibit the growth of neighboring commensal bacteria using reutericyclin, an acylated tetramic acid produced by the *muc* biosynthetic gene cluster. Here, a complete genome sequence of B04Sm5 is reported.

## Abstract (precis)

Streptococcus mutans strain B04Sm5 was recently described as utilizing reutericyclin, an acylated tetramic acid produced by the *muc* biosynthetic gene cluster, to inhibit the growth of neighboring commensal bacteria. Here, a complete genome sequence of B04Sm5 is reported.

## ANNOUNCEMENT

Streptococcus mutans is considered a major etiologic agent of dental caries, which is globally the most common chronic infectious disease (1). As it is not typically considered a pioneer colonizer of the tooth, S. mutans must be able to outcompete its already-established bacterial neighbors (which are typically health-associated commensals) to successfully institute itself as a member of the dental plaque microbiota and cause disease (2, 3). A subset of S. mutans strains (but not the well-characterized type strain, UA159) encode a hybrid nonribosomal peptide synthetase/polyketide synthase (NRPS/PKS) biosynthetic gene cluster (BGC), *muc*, which has significant homology (48% to 69%) to cognates involved in the biosynthesis of reutericyclin by *Lactobacillus* (4–7). Indeed, the S. mutans B04Sm5 *muc* BGC was recently shown to produce not only reutericyclin but also two reutericyclin analogs, as well as mutanocyclin, an unacylated reutericyclin derivative (6). S. mutans B04Sm5 employed reutericyclin to inhibit the growth of the adjacent competing commensal organisms, Streptococcus sanguinis, Streptococcus mitis, and Streptococcus gordonii (6). This suggests that the carriage of *muc* by S. mutans strains may impact the virulence of this pathogen.

S. mutans B04Sm5, a serotype c strain, was originally isolated from a carious lesion in a child with rampant caries by Argimon and Caufield, as described in reference 8. A draft assembly of the genome of B04Sm5, fragmented into 81 contigs, was published in 2014 (9). Here, a combination of Oxford Nanopore and Illumina sequencing was used to obtain a complete, circular genome sequence. B04Sm5 was grown in brain heart infusion (BHI) medium at 37°C under 5% CO_2_/95% air. High-molecular-weight genomic DNA (gDNA) was extracted using a phenol-chloroform-based protocol (10) with the following modifications: (i) 300 U/ml of mutanolysin was used in lysis; (ii) following lysis in SDS, the sample was homogenized using 0.1-mm glass beads for 30 s in a FastPrep-24 homogenizer (MP Biomedicals); and (iii) an additional final ethanol precipitation was performed. The resulting gDNA was examined for purity, size, and concentration using the TapeStation system (Agilent Technologies) and a Qubit fluorometer (Thermo Fisher Scientific). A long-read library was prepared using a ligation sequencing kit (Oxford Nanopore Technologies) and sequenced on the GridION system using an R9.4.1 flow cell (Oxford Nanopore Technologies). Base calling was performed using Guppy v.4.8.11, resulting in 780,164 reads (*N*_50_, 14,642 bp). A short-read library was prepared using a TruSeq DNA PCR-free library prep kit (Illumina) and sequenced on a MiSeq instrument (Illumina), generating 486,071 paired-end 150-bp reads. Quality control was performed on the short reads using KneadData v.0.5.4 (https://github.com/biobakery/kneaddata) and on the long reads by Filtlong v.0.2.0 (https://github.com/rrwick/Filtlong). Draft assemblies were produced using a combination of the long- and short-read libraries and the Flye v.2.8-b1674 (11), hybridSPAdes v.3.14.0 (12), and Unicycler v.0.4.8 (13) assemblers. Trycycler v.0.3.0 (https://github.com/rrwick/Trycycler) was used to develop a circular consensus assembly from the draft assemblies. The final assembly was polished using the full long-read set with medaka v.1.0.3 (https://github.com/nanoporetech/medaka) and then the short-read set using Pilon v.1.23 (14). The scripts and parameters of the software tools used are available at https://github.com/jaybake5/B04Sm5_genome_completion. The genome of B04Sm5 is a single chromosome of 2,011,542 bp, encoding 1,964 genes with a GC content of 36.9%. This resource will provide valuable information regarding the acquisition/evolution of reutericyclin and mutanocyclin production in S. mutans.

### Data availability.

The complete genome sequence of S. mutans B04Sm5 has been deposited in GenBank under the accession number CP061071. The BioProject accession number for the genome is PRJNA661123. The raw read libraries (both Oxford Nanopore and Illumina) have been deposited in the Sequence Read Archive (SRA) database under the accession number PRJNA661123.
